# Posttraumatic stress symptoms and mental health services utilization in adolescents with social anxiety disorder and experiences of victimization

**DOI:** 10.1007/s00787-012-0336-z

**Published:** 2012-10-26

**Authors:** Malin Gren-Landell, Nikolas Aho, Elisabeth Carlsson, Annica Jones, Carl Göran Svedin

**Affiliations:** 1 The Child and Adolescent Psychiatric Clinic, The University Hospital of Linköping, S-581 85 Linköping, Sweden; 2 The Child and Adolescent Psychiatry, Department of Clinical and Experimental Medicine, Faculty of Health Sciences, Linköping University, Linköping, Sweden; 3 Department of Behavioural Sciences and Learning, The Swedish Institute for Disability Research, Linköping University, Linköping, Sweden

**Keywords:** Social anxiety disorder, Victimization, Adolescents, Posttraumatic stress, Mental health utilization

## Abstract

Recent findings from studies on adults show similarities between social anxiety disorder (SAD) and posttraumatic stress in the form of recurrent memories and intrusive and distressing images of earlier aversive events. Further, treatment models for SAD in adults have been successfully developed by using transdiagnostic knowledge on posttraumatic stress symptoms (PTSS). Studies on adolescents are though missing. The present study aimed at exploring the association between PTSS and SAD in Swedish adolescents. A second aim was to study mental health services utilization in relation to these conditions. A total of 5,960 high-school students participated and reported on SAD, life time victimization, PTSS and mental health service utilization. Socially anxious adolescents reported significantly higher levels of PTSS than adolescents not reporting SAD and this difference was seen in victimized as well as non-victimized subjects. Contact with a school counselor was the most common mental health service utilization in subjects with SAD and those with elevated PTSS. In the prediction of contact with a CAP-clinic, significant odds ratios were found for a condition of SAD and elevated PTSS (OR = 4.88, 95 % CI = 3.53–6.73) but not for SAD only. Screening of PTSS in adolescents with SAD is recommended. The service of school counselors is important in detecting and helping young people with SAD and elevated PTSS. Clinical studies on SAD and PTSS in adolescents could aid in modifying treatment models for SAD.

## Introduction

Social anxiety disorder (SAD) is a common mental disorder [1] with typical onset during adolescence [2]. Among other risk factors, various kinds of negative life events are proposed to contribute to the onset and maintenance of SAD [2]. Retrospective studies show higher rates of childhood maltreatment, physical abuse and sexual abuse in adults with SAD compared to non-socially anxious subjects [3–7]. In concurrent studies on children and adolescents, peer victimization is reported to be associated with SAD [8, 9]. Studies on other forms of victimization and SAD in young people are still sparse though [10].

Posttraumatic stress disorder (PTSD) is mainly thought of as an effect of victimization on mental health [11]. SAD co-occurs with PTSD [12] as reported mainly in adult combat veterans [13–15]. The association between SAD and PTSD is less well studied in children and adolescents [16]. However, Pynoos and colleagues [17] emphasize a model within the paradigm of developmental psychopathology where pediatric anxiety disorders and reactions to traumatic events interplay in exacerbation of posttraumatic stress symptoms (PTSS) and for example avoidance behaviour typical of SAD [17]. There is support for anxiety disorders, and especially SAD, as a significant predictor of PTSS and PTSD [18, 19]. Copeland [18] reported an increased risk of experiencing traumatic events in individuals with SAD and that SAD predicted PTSD but not vice versa.

A diagnosis of PTSD requires exposure to a life threatening event which constitutes the A1 criterion [11]. Interestingly, there are findings that socially phobic individuals perceive social events as more distressing than life-threatening events that constitute the A1 criterion of PTSD [20, 21]. Erwin and colleagues [20] found that individuals with SAD reacted with PTSS like re-experiencing, avoidance and hyperarousal in relation to memories of socially stressful events. A similar finding was reported by Carleton and colleagues [21] where subjects that had experienced a negative social event reported higher levels of social anxiety and PTSS than those reporting only criterion A1 events. In fact, theoretical guidance for the understanding and treatment of SAD can be offered by studying shared mechanisms of SAD and PTSD [22]. Common to both conditions is the occurrence of intrusive and distressing emotional images and attempts to avoid these [22]. Studies on imagery, i.e., mental images, have contributed to the development of effective interventions for both conditions in adults [23] and changing imagery has shown promising results especially in the case of social anxiety [24, 25]. However, there are no published studies on adding these components in treatment for SAD in adolescents.

Not only is development of treatment models of interest due to new empirical findings but also due to low rates of mental health utilization in SAD [26–28]. Rates of help-seeking and mental health utilization in children and adolescents with PTSD are less well known than for SAD [29]. In adults, strong correlations have been reported between severity of PTSS and the likelihood of seeking help from a mental health professional [30]. Though there is little theoretical guidance on barriers and facilitators of help-seeking in SAD and in PTSD, it is possible that a condition of SAD may be perceived as a trait and unchangeable temperament that does not give rise to help-seeking [31]. Symptoms like re-experiencing on the other hand may be frightening and cause contact for professional help as the experience of distress in adolescents is associated with help-seeking [32]. The opposite is also possible, i.e., fear of being perceived as crazy when telling about posttraumatic symptoms may constitute a barrier. In order to find facilitators of mental health utilization for SAD as well as for PTSD, studies on predictors are needed.

## Aim

According to the findings of Erwin et al. [20] i.e., social events being perceived as more distressing than criterion 1A events of PTSD, we choose to study PTSS in the present study. The main aim of the study was to explore reports of PTSS in adolescents with SAD. The study was part of a survey on prevalence of victimization in Swedish adolescents. We expected that adolescents reporting SAD would show higher levels of victimization and of PTSS than those not reporting SAD. Partially due to the cross-sectional design of the study we also expected an inverse relation of SAD in those reporting elevated PTSS.

The second aim was to describe use of mental health services in subjects with SAD and in those with elevated levels of PTSS. Analyses were conducted separately for males and females as gender differences exist in prevalence of SAD [1] and posttraumatic stress [33] as well as in mental health service utilization for anxiety disorders [33, 34].

## Methods

### Participants

In order to obtain a representative sample, sampling was conducted in reference to a division of Sweden’s municipalities based on structural characteristics including population size, commuting patterns and economic structure. Five percent of all Swedish students in their second year of high-school studies were the goal for the sampling procedure. Municipalities of different sizes were selected to control for a potential confounding effect of municipality-size: large (>200,000 inhabitants), middle-sized (50–200,000 inhabitants) and small municipalities of <25,000 inhabitants.

In total, students from 51 high-schools participated. There were 5,960 (49.6 % females, 50.4 % males) of 7,849 eligible students who participated, which meant a response rate of 75.9 %. The participants were aged 16–20 years (*M* = 17.3, SD = .64). In 16.8 % of the students both parents were born outside Sweden. The sample was considered representative of the population when comparing data on ethnicity and gender with national statistics. Also, paternal unemployment (4.7 %) was similar to the rate in Sweden at the time for the survey. The proportion of adolescents living in a small municipality were 17.1 % (*n* = 1,017), 48 % (*n* = 2,858) in a middle-sized municipality and 35 % (*n* = 2,085) in a large municipality.

### Procedure

Participation was voluntary and anonymous. The students were informed about this before the survey took place by letter via teachers and by means of a written description in the classroom at the time of the survey. Students who wanted to participate were informed to show up at the room prepared for the survey and were informed that they could cancel participation at any time. Active participation was considered informed consent. Further, the students were given instructions about where to get counselling if participation caused feelings of distress. The study protocol was approved by the ethics committee at Linköping University.

Questionnaires were computerized (portable PC or via a web browser) and the students completed these during school-time. The project manager was present during the whole survey to answer any questions. All participants were asked to respond to all items and items could not be skipped. The participants received a voucher for a movie ticket once they had completed or cancelled the survey.

### Measures

#### The Social Phobia Screening Questionnaire for Children (SPSQ-C)

The SPSQ-C is a modified version of the Social Phobia Screening Questionnaire (SPSQ), adapted and validated for use with children and adolescents [35]. A psychometric evaluation of the SPSQ-C showed a test–retest reliability of *r* = .60. When compared to diagnostic structured interview a specificity of 86 % and a sensitivity of 71 % were found [36]. The SPSQ-C is based on the diagnostic criteria of SAD, also called social phobia, in the DSM-IV [11]. Eight items cover eight social potentially phobic situations like “speaking in front of the class”, “raising your hand during a lesson”, “looking someone in the eyes during a conversation”. On the initial item the participant rates fear of each of the eight situations on a scale ranging from 1 (*No fear*) to 3 (*Marked fear*). This item represents the criterion A. Next follow five items covering criteria A of the DSM-IV (fear that others will notice that I am nervous), criteria B (find these situations distressing) and criteria D (try to avoid these situations) for one or more of the phobic situations. Since the youths were below 18 years of age the C-criteria, realizing that the fear is excessive or unreasonable, did not have to be fulfilled. The seventh item assess criterion E by three yes/no questions, i.e., the student was asked whether the social fear was of such nature that it severely interfered with his/her activities in school, during leisure-time or when being with peers. The eighth and last question covered the F-criterion of 6-month duration (yes/no question). In the present study, internal consistency was measured for the first item covering eight phobic situations and alpha = .83.

In order to fulfil a diagnosis of SAD based on the responses on the SPSQ-C, i.e., a probable case of SAD, the respondent had to rate at least one of the eight potentially phobic situations as “marked fear”. This particular situation had to be consistently endorsed in the diagnostic questions covering social phobia criteria. Thus, a categorical measure was applied. The cut-point used was based on fulfilling the DSM-IV criteria of SAD or not.

#### The Juvenile Victimization Questionnaire (JVQ)

The JVQ [37] consists of 34 screening items on offenses against young people and covers five domains: conventional crime (e.g., robbery, vandalism, assault with or without weapon); maltreatment (e.g., physical or psychological abuse by caregiver and neglect); peer or siblings victimization (e.g., assault from gang or from sibling, biased attacked, physical or emotional bullying); sexual victimization (e.g., assault by known or unknown adult or peer, non-specific sexual assault, rape, flashing/sexual exposure, verbal sexual harassment) and witnessing victimization (e.g., domestic violence, burglary of family household, murder of family member or friend, exposure to combat or ethnic conflict). In the present study α = .83 for all items, for conventional crime: α = .66, sexual victimization: α = .64, maltreatment: α = .55, peer/sibling victimization: α = .52 and for witnessing victimization α = .51.

The JVQ can be used in a self-administered format from the age of 12, which was the case in the present study. The self-administered format has proven to have good test–retest reliability and construct validity [38]. The participants reported on victimization during the prior year, and victimization before the prior year. In the data analysis, a lifetime scale was created that summed up the items from before prior year and during prior year. This was due to the criteria of 6 months duration of social anxiety to establish a diagnosis of SAD [11] and accordingly data on prior year was of minor interest.

The JVQ is in part built on American legal and insurance issues. In the present study, an adaptation was made in the Swedish version, removing item 25 (“In the last year/before last year, did you do sexual things with anyone 18 or older, even things you both wanted?”). This was due to a marked difference between Swedish and the American legal system making the item irrelevant for Swedish conditions. This means that 33 items were used. Due to technical problems an internal attrition occurred on item 3 and 5 of the subscale “conventional crime”. A comparison of the data in the attrition group and the total sample showed no significant differences regarding prevalence of SAD or PTSS or on reporting mental health utilization, but there was a significantly higher proportion of females than males in the attrition group (χ^2^ = 21.79, *df* = 1, *p* < .001) compared to the total sample.

The respondent rated if an event was experienced and if so, the number of times each event had been experienced (i.e., 1 time, 2–5 times, 6–10 times, >10 times or no times). For the data analysis, each item was dichotomized into not victimized or victimized. A total score of each domain was obtained for the whole scale and the subscales, respectively.

#### The Trauma Symptom Checklist for Children (TSCC)

The TSCC is a self-report questionnaire that measures trauma-related symptoms in children and adolescents [39]. Psychometric data are reported on Swedish adolescent populations [32]. The questionnaire consists of 54 items divided into six subscales: Anxiety, Depression, Posttraumatic stress (PTS), Anger, Sexual concerns and Dissociation. In the present study, only data from the PTS-scale was used. The items of the PTS-scale concern nightmares, remembering scary things, difficulty stopping intrusive thought about bad things that have happened, scary ideas or pictures pop into the head etc. Each item was answered with the following alternatives: *never, sometimes, often and almost always.* Internal reliability for the PTS-scale in the present study was α = .86.

#### Sociodemographic variables

A range of socio-demographic data were collected but only data on age, gender, birth of origin (i.e., Swedish or not Swedish) and urban status are reported.

#### Mental health utilization

Items on mental health utilization were administered. These were answered by *yes* or *no.* The following items were included: *Have you during life time been in contact with…School psychologist; School counselor; Social worker; Child and Adolescent Psychiatry (CAP); Children’s Rights in Society; Else; None of the above alternatives.* Respondents were not asked about the reason for having been in contact with any of the above services, or when, or how many times. They were also asked if they ever had used medication for being depressed, anxious or having seeping difficulties or problems with hyperactivity. Data on use of social worker and children’s rights in society were not part of the objective of this paper.

### Data analysis

Demographic variables were compared between those with SAD and those without SAD using cross-tabulations with Chi-square statistics. The oldest age group was composed of those of age 19 years and of 20 years as there were too few subjects with SAD (*n* = 3) to use cross-tabulation. Group-differences between subjects reporting SAD and subjects without SAD due to victimization and PTSS were analysed by independent *t* tests. A recommended cut-off score of 13 for females and 10 for males was used to identify subjects with elevated PTSS [33]. Binary logistic regression was used to predict mental health utilization from the following independent variables: SAD only, elevated PTSS only and comorbid SAD and elevated PTSS, controlling for sociodemographic variables. All statistical analyses were performed using the SPSS 18.0 software package.

## Results

### Sociodemographic data

Self-reported SAD was found in 10.2 % (*n* = 605) of the total group (*n* = 5,960). A significant gender difference emerged with more females than males reporting SAD. Prevalence rates increased with increasing age and prevalence of SAD differed significantly due to ethnicity with higher rates in those with Swedish birth of origin. Also higher rates were seen in adolescents from small and middle-sized municipalities compared to large municipalities. See Table 1 for all data.

**Table 1:** Prevalence of SAD and of elevated PTSS in relation to sociodemographic variables

	SAD	No SAD		Elevated PTSS	No elevated (PTSS)	
	% (*n*)	(*n*)	χ^2^	% (*n*)	(*n*)	χ^2^
Gender						
Male	5.8 (173)	2,829		13.3 (399)	2,603	
Female	14.6 (432)	2,526	128.39***	16.4 (484)	2,474	11.14***
Age (years)						
16	8.9 (27)	277		12.5 (38)	266	
17	9.9 (384)	3,481		14.1 (545)	3,320	
18	10.1 (156)	1,389		15.3 (236)	1,309	
19–20	15.4 (38)	208	8.30*	26.0 (64)	182	27.57***
Ethnicity (child)						
Non-Swedish	6.0 (32)	500		20.5 (109)	423	
Swedish	10.6 (573)	4,855	10.96***	14.3 (774)	4,654	14.90***
Municipality						
Small	11.2 (234)	1,851		15.3 (318)	1,767	
Middle	10.2 (292)	2,566		14.7 (419)	2,439	
Large	7.8 (79)	938	8.97**	14.4 (146)	871	.54^ns^

* *p* < .05

** *p* < .01

*** *p* < .001

Elevated PTSS, i.e., above clinical cut-off on the PTS-scale of the TSCC, was found in 14.8 % (883) and significantly higher rates were seen in females compared to males subjects with non-Swedish birth of origin compared to those with Swedish origin and among those in age 19–20 years compared to the younger adolescents. See Table 1.

### SAD, victimization and PTSS

In the total group the mean value of lifetime victimization was 4.12 (SD = 4.04). Subjects with self-reported SAD reported significantly higher rates of victimizing experiences compared to the no-SAD group as well as higher levels of PTSS on the PTS-scale of the TSCC, (see Table 2 for all data). Within the SAD-group no significant differences were found between genders regarding life time victimization (*t* = 1.53, *df* = 535, ns) but significantly higher levels of PTSS were found in females with SAD compared to males with SAD (*t* = 5.55, *df* = 603, *p* < .001).

**Table 2:** Reports of PTSS and of victimization in subjects with SAD and without SAD

	SAD (*n* = 605)	No SAD (*n* = 5,355)	
	*M* (SD)	*M* (SD)	*t* test
PTSS	9.77 (5.68)	5.94 (4.92)	15.92***
Females	10.56 (5.62)	7.33 (4.92)	11.25***
Males	7.79 (5.36)	4.70 (4.58)	7.42**
Victimization	5.54 (4.79)^a^	3.96 (3.92)^b^	7.36***
Females	5.74 (4.92)	4.22 (4.05)	5.71***
Males	5.04 (4.41)	3.74 (3.80)	3.56***

** *p* < .01

*** *p* < .001

^a^ *n* = 68 (20 males and 48 females)

^b^ *n* = 560 (241 males and 319 females)

### SAD and elevated PTSS

**Fig. 1:**
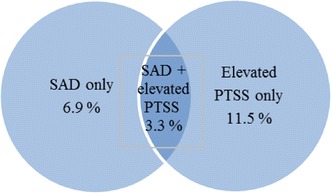
Prevalence of SAD only, cases with elevated PTSS only and cases with both SAD and PTSS in the total sample

When studying each condition separately, reports of SAD only were more common (*n* = 410) than reporting both conditions (*n* = 195) and most common were reports of only elevated PTSS (*n* = 688), see Fig. 1.

Elevated PTSS was found in 32.2 % (*n* = 195) of adolescents reporting SAD compared to 12.8 % (*n* = 688) in those not reporting SAD. In the SAD-group, no gender differences were found for clinical levels of PTSS. Clinical levels of PTSS were reported by 32.9 % (*n* = 57) of males with SAD and by 31.9 % (*n* = 138) of females.

The inverse relation showed that 22.1 % of adolescents with SAD reported elevated PTSS (14.3 % among males; 28.5 % among females).

### Mental health utilization

The most common service use was contact with a school counselor as reported by 26.7 % of the total sample. Significantly higher rates of utilization of all investigated forms of services were seen in those with self-reported SAD compared to those not reporting SAD. For example, among subjects with SAD, 18.3 % (*n* = 111) reported having had contact with a CAP-clinic during lifetime compared to 9.1 % of subjects without SAD (χ^2^ = 51.30, *df* = 1, *p* < .001). However, when comparing the group of subjects reporting SAD only and not elevated PTSS, lower rates of mental health utilization were seen in those with SAD only compared to those with elevated PTSS or both conditions, see Table 3 for all data. Significant gender differences were found for all services except contact with school psychologist in the group of elevated PTSS but no significant differences for any services were found in the group of both conditions.

**Table 3:** Use of mental health services during life time among those with SAD only (*n* = 410), only elevated PTSS (*n* = 688) and those with both SAD and elevated PTSS (*n* = 195). Percentages within each group and within each gender

	SAD	Elevated PTSS	SAD + elevated PTSS
	(%)	(%)	(%)
Child psychiatric service	11.5	17.4	32.8
Female/male	11.6/11.2	23.7/11.1**	36.2/24.6
Psychotropic medication	8.0	18.2	28.2
Female/male	8.8/6.0	23.1/13.2**	31.9/19.3
School counselor	33.9	43.6	53.8
Female/male	37.4/25.0*	52.6/34.5**	58.0/43.9
School psychologist	7.8	15.6	23.1
Female/male	8.8/5.2	16.8/14.3	24.6/19.3

Figures in bold represent significant differences between genders

* *p* < .05

** *p* < .001

The highest odds ratios for having had contact with a CAP-clinic was found for the combined group of SAD and elevated PTSS (OR = 4.88, 95 % CI = 3.53–6.73, *p* < .001), followed by the group of elevated PTSS and no SAD (OR = 2.50, 95 % CI = 2.00–3.14, *p* < .001). However, no significant prediction was made from the group of subjects with SAD but not elevated PTSS (OR = 1.26, 95 % CI = .91–1.74). Significant associations were also found between use of CAP-clinic service and female gender (OR = 2.05, 95 % CI = 1.70–2.45), Swedish birth of origin (OR = 1.68, 95 % CI = 1.16–2.42) and living in a small municipality (OR = 1.33, 95 % CI = 1.02–1.73).

## Discussion

In recent years, the co-variation between SAD and PTSS has been explored in adult populations [19]. However, until now studies on SAD in relation to victimization and to PTSS in adolescents have been missing. The following results thus represent first preliminary findings and can be summarized according to the following.

Firstly, significantly higher rates of life time victimization were found in socially phobic adolescents compared to those not reporting SAD. Besides, socially distressing experiences were not measured in the present study and thus rates of potentially traumatic events may be even higher. Theoretical models [17] and empirical findings [19] suggest an increased risk of experiencing traumatic events as well as reacting with PTSD in individuals with phobic disorders like SAD.

Secondly, significantly higher levels of PTSS were found in socially phobic adolescents compared to those not presenting with SAD. One-third of males with SAD and the same proportion of females reported clinical levels of PTSS. As expected, an inverse relation was also found though gender differences emerged in this respect. Looking at the inverse relation, lower rates of SAD were found among males than among females, which can be explained by the lower prevalence of SAD among males as whole. Analyses of the time-related associations of SAD preceding PTSS or vice versa were though not possible to conduct in the present study. The finding indicates use of screening of SAD in cases of PTSS and vice versa.

In the present study, all data were analysed in relation to gender. In the SAD-group similar rates of victimization were found for both genders but significantly higher rates of PTSS. Other studies show that females react with more symptoms in relation to trauma and that females are more exposed to certain forms of victimization, like sexual abuse, that cause more symptoms [40]. Contrary to that result, no gender differences emerged concerning elevated PTSS in the SAD-group. Thus, attention to victimization and PTSS in males seems warranted.

In the total sample, 3.3 % reported SAD and elevated PTSS. This finding indicates that for a subgroup of adolescents with SAD, treatment models of SAD may need to include components that address PTSS as has been successfully demonstrated in adults. Posttraumatic symptoms like intrusive memory images involve emotions and physical sensations in the same way as if being confronted by an anxiety eliciting situation. Memory images like these commonly occur in persons suffering from SAD and the images are often based on prior social experiences that the person has experienced as negative and anxiety provoking [23]. Further, on these images become more and more negative and distorted in conjunction with the critical self-image common in persons with SAD. The images are updated in situations reminding of the unpleasant social situations. Addressing the unifying characteristics and components commonly seen in the treatment of PTSD could thus be of value in the treatment of SAD like exposure not only to real events but also to memories of past events [41]. However, this method has not been empirically evaluated in adolescent populations.

The development and evaluation of new treatment methods are only of value if mental health services are used by those in need of them and the second aim of the present study was to investigate mental health services utilization in cases of SAD and cases of clinical levels of PTSS. The highest odds ratios for having been in contact with a CAP-clinic during life time were found for those reporting PTSS and SAD. A condition of SAD did not significantly predicted contact with a CAP-clinic. Among the socially phobic adolescents less than 1/5 had used child psychiatric services during life time. This rate is in concordance with prior studies [26, 27]. Among adolescents with SAD who did not report elevated PTSS only 11 % had been in contact with a CAP-clinic. Thus, it seems like adolescents with SAD can “get a little help” from clinical levels of PTSS to use child psychiatric services.

However, in all groups the most common use of mental health service was in contact with a school counselor. In Sweden, children and adolescents can get in contact with this service without the help of parents or other adults which make access easier than to a school psychologist or a CAP-clinic. Adolescents are more willing to seek help for mental health problems in the presence of established and trusted relationship with potential help providers [34, 42]. Appraisal of a problem as something to seek help for is reported as another predictor for help-seeking [34]. In the study of Colognori [28], disclosure of social anxiety to school personnel was associated with access to treatment and by a shorter time interval. Thus, help-seeking may be facilitated by that school counselors have good knowledge on SAD and PTSS and that they inform students that these are conditions that are recognized and can be treated.

## Limitations

The present study constitutes a large and representative study on SAD and PTSS in adolescents. The main limitation is the use of a cross-sectional design precluding conclusions on a causal relationship. Further, investigation of victimization was not specifically designed to measure socially distressing events as may be relevant for a condition of SAD. Still, JVQ aims at measuring interpersonal events and as such constitute a valid instrument for the objective of the study. Another limitation is that the participants were not asked about the reasons for mental health utilization. Other reasons than SAD or PTSS, such as depression or other anxiety disorders, may have accounted for the mental health contact but was not controlled for in the present study. Commonly, comorbid conditions constitute the reason for help-seeking and it is probable that SAD would not have been seen as the reason for mental health utilization. Most children and adolescents do not refer themselves to CAP and commonly neither parents [43] nor teachers [44] recognize social anxiety as a problem in children.

## Conclusions

Clinical levels of PTSS are seen in adolescents with SAD and this comorbid condition is associated with higher rates of mental health utilization than in cases of SAD without elevated PTSS or elevated PTSS without SAD. The relationship between SAD and PTSS has not been well recognized and needs to be explored in well controlled studies on clinical samples. The results point to including questions on PTSS in the assessment of SAD. Further, treatment models for SAD in adolescents need to take into account the occurrence of PTSS that may help by providing these individuals with a rationale to better understand and cope with their symptoms. The results also imply that if PTSS are indicated, screening of SAD can be of value.
